# Insights on the Electrocatalytic Seawater Splitting at Heterogeneous Nickel-Cobalt Based Electrocatalysts Engineered from Oxidative Aniline Polymerization and Calcination

**DOI:** 10.3390/molecules26195926

**Published:** 2021-09-30

**Authors:** Perla Hajjar, Marie-Agnès Lacour, Nathalie Masquelez, Julien Cambedouzou, Sophie Tingry, David Cornu, Yaovi Holade

**Affiliations:** 1 Institut Européen des Membranes, IEM, UMR 5635, University Montpellier, ENSCM, CNRS, 34090 Montpellier, France; perla.hajjar@enscm.fr (P.H.); nathalie.masquelez@umontpellier.fr (N.M.); julien.cambedouzou@enscm.fr (J.C.); sophie.tingry@umontpellier.fr (S.T.); david.cornu@enscm.fr (D.C.); 2 ChemLab, ENSCM, 34296 Montpellier, France; marie-agnes.lacour@enscm.fr

**Keywords:** polyaniline, electrocatalysis, hydrogen evolution reaction, oxygen evolution reaction, seawater splitting

## Abstract

Given the limited access to freshwater compared to seawater, a growing interest surrounds the direct seawater electrolysis to produce hydrogen. However, we currently lack efficient electrocatalysts to selectively perform the oxygen evolution reaction (OER) over the oxidation of the chloride ions that are the main components of seawater. In this contribution, we report an engineering strategy to synthesize heterogeneous electrocatalysts by the simultaneous formation of separate chalcogenides of nickel (NiS_x_, x = 0, 2/3, 8/9, and 4/3) and cobalt (CoS_x_, x = 0 and 8/9) onto a carbon-nitrogen-sulfur nanostructured network. Specifically, the oxidative aniline polymerization in the presence of metallic cations was combined with the calcination to regulate the separate formation of various self-supported phases in order to target the multifunctional applicability as both hydrogen evolution reaction (HER) and OER in a simulated alkaline seawater. The OER’s metric current densities of 10 and 100 mA cm^−2^ were achieved at the bimetallic for only 1.60 and 1.63 V_RHE_, respectively. This high-performance was maintained in the electrolysis with a starting voltage of 1.6 V and satisfactory stability at 100 mA over 17 h. Our findings validate a high selectivity for OER of ~100%, which outperforms the previously reported data of 87–95%.

## 1. Introduction

The transition from a fossil fuel-based society to less energy-intensive processes with low environmental impact motivates pressing actions at several scales [1]. Among the various possibilities, H_2_ is a cornerstone for the energy transition. It acts as an energy carrier in fuel cells, a reagent of Haber-Bosch process for the production of ammonia (needed in the nitrogen fertilizer industry), and a fuel in metallurgy among others. The main production pathways (95%) by methane reforming and coal gasification not only generate CO_2_ pollution (which is no longer a decarbonized pathway) but also require high operating temperatures (and thus a significant energy input) [1,2,3]. Moreover, the resulting H_2_ must be cleaned of carbon impurities before use, which adds complexity and costs. Electrochemical converters can not only synthesize H_2_ of high purity without CO_2_ by using renewable electricity (hydroelectricity, wind, solar …) but also use it to produce electricity via reversible fuel cells and electrolyzers (2H_2_O = 2H_2_ + O_2_) [4,5]. Significant advances have demonstrated that different electrocatalytic materials can enable water electrolysis in acidic or alkaline media closely at its thermoneutral cell voltage of 1.45 V [6,7]. However, a crucial societal question surrounds the race towards green H_2_ and the acceptance of the hydrogen economy: What water should be used to produce H_2_? While the use of “pure water” is trivial for obtaining high-performance at a noble metal-free (Ni, Co, Fe, etc.) electrocatalysts [8,9,10,11,12,13,14,15,16], there is a growing concern about the water supply for H_2_ electrolyzers [3,17,18,19,20]. It is argued that the access to this resource is already problematic and thus requiring large quantities of freshwater might become a concern if the water electrolysis technology is deployed more widely in the hot and arid regions that have very limited access to freshwater but significant access to seawater [19,20,21]. Indeed, the freshwater is only 1% of the planet’s water and all remaining 99% or ca. 1.4 billion km^3^ is composed of seawater [18,22].

The emerging solution is to directly fuel the electrolyzers with seawater to avoid additional purification and energy requirements. However, this is challenging given the pretty complex nature of the seawater. Indeed, the dissolved ions can poison, react to, or accelerate the degradation of the electrolyzer components (membrane, catalysts, etc.) by the formation of soluble species at both cathode and anode [21,22,23]. NaCl being the main constituent (average content of 35 g per L), the roadmap is to test the possible electrocatalysts using seawater-based electrolyte, which consists of running the experiments in the absence and presence of NaCl (0 to 3 M) [23,24,25,26,27,28,29,30,31,32,33,34], similar to studies in corrosion science [35]. Vos et al. [28] have examined the likely competition between the oxygen evolution reaction (OER) and the chlorine evolution reaction at the anode (CER, Equation (1)), which is known as the chloride ions oxidation (ClOR, Equation (2)). They found that a heterogeneous electrocatalyst MnO_x_/IrO_x_ reduces the CER selectivity from 86% to less than 7%. Given the toxicity of Cl_2_ gas with corrosive properties (even though it is one of the key industrial chemicals) and the oxidative ability of the mixture “NaCl + NaClO”, the electrolysis of seawater for H_2_ should favor OER (Equation (3)) instead of CER or ClOR (O_2_ can be vented without any caution). Recent results show that the alkaline medium (the most envisaged media [36,37]) produces the best selectivity at Ni-based electrocatalysts (200 mA cm^−2^ and 1.6 V for 100 h) [23,25,26,27,38]. Amikam et al. [39] found that ClOR in NaCl-saturated solutions is inhibited at [NaOH] ≥ 2.5 M. This is particularly interesting for real electrolysis conditions where NaCl may accumulate in the electrolyte if seawater is continuously fed to the system and H_2_O is converted to H_2_ and O_2_ [31,32]. Less research is dedicated to that while the continuous neutral seawater electrolysis can also likely trigger the chloride ions accumulating progressively to accelerate the corrosion processes and/or to favor undesirable chloride oxidation (ClOR) to chlorine/hypochlorite [31]. However, it is expected to operate under more realistic conditions in order to suppress the “valley of death” between the fundamental and applied research in electrolysis [40].

While, thermodynamically, the Pourbaix diagrams show that OER is favored over ClOR (Equation (4)) [21,27], it is kinetically much slower because of the four-electron transferred process that generates many reaction intermediates and activation energy barriers (two-electron for ClOR with likely one intermediate). Therefore, without an efficient design, there could be an inversion of the selectivity from a given potential. Moreover, the engineering of the anode electrocatalyst is often different from that of the cathode [29], which complicates the electrolyzer assembly. Therefore, the use of a multifunctional material at both cathode (HER) and anode (OER) is greatly desired to simplify the design of new generation electrolyzers and possibly contribute to their cost reduction.

CER: 2Cl^−^ → Cl_2_ + 2e^−^, E°(Cl_2_/Cl^−^) = (1.36 + 0.059×pH) V_RHE_, highly acidic pH, (1)

ClOR: Cl^−^ + 2OH^−^ → ClO^−^ + H_2_O + 2e^−^, E°(ClO^−^/Cl^−^) = 1.72 V_RHE_, pH > 7, (2)

OER: 4OH^−^ → O_2_ + 2H_2_O + 4e^−^, E°(O_2_/OH^−^) = 1.23 V_RHE_, pH > 7, (3)

HER: 2H_2_O + 2e^−^ → H_2_ + 2OH^−^, E°(H_2_O/H_2_) = 0 V_RHE_, pH > 7, (4)

E_ClOR_ − E_OER_ = 1.72 − 1.23 = 0.49 V, pH > 7, (5)

The goal of the present study is to examine the potentiality of heterogeneous nickel-cobalt based electrocatalysts engineered from the oxidative aniline polymerization and calcination for dual OER and HER in alkaline media in the presence of sufficient NaCl to mimic the seawater electrolysis (herein, 1 M instead of the typical case of 0.5 M). The chemical polymerization in the presence of Ni^2+^ and Co^2+^ plus calcination at 900 °C under N_2_ has enabled to develop self-supported nickel and cobalt chalcogenides onto a carbon-nitrogen-sulfur nanostructured network. This procedure differs from conventional procedures where the metal nanoparticles are initially prepared before the use of carbon black to lower the metal content and do not allow an optimal operation.

## 2. Experimental Methods

### 2.1. Materials and Chemicals

Sodium chloride (NaCl, ≥99.5%(AT) ACS, Sigma Aldrich, St. Louis, MO, USA), nickel (II) nitrate hexahydrate (Ni(NO_3_)_2_·6H_2_O, 99%, Acros Organics, Geel, Belgium), cobalt (II) nitrate hexahydrate (Co(NO_3_)_2_·6H_2_O, ACS, 98.0–102.0%, Alfer Aesar, Haverhill, MA, USA), aniline (ANI, 100%, Alfa Aesar), hydrochloric acid (HCl, 37%, VWR, Radnor, PA, USA), ammonium persulfate ((NH_4_)_2_S_2_O_8_, APS, 98%, Merck, Kenilworth, NJ, USA), isopropanol (iPrOH, 99.5%, Sigma Aldrich), sodium thiosulphate pentahydrate (Na_2_ S_2_O_3_∙5H_2_O, 99%, VWR), Nafion^®^ suspension (5 wt%, Sigma Aldrich), and sodium hydroxide (NaOH, 99.4%, Fisher Scientific, Hampton, NH, USA) were used as-received. Gas diffusion electrode as carbon paper (AvCarb MGL370, 370 μm thickness) was obtained from Fuel Cell Earth LL (Stoneham, MA, USA). Pt/Vulcan (20 wt %, 2–3 nm) was purchased from Premetek Co., Cherry Hill, NJ, USA. Ultrapure water (18.2 MΩ cm at 20 °C) was produced from a Milli-Q Millipore (Burlington, MA, USA) source.

### 2.2. Synthesis of Heterogeneous Electrocalysts

We have utilized the oxidative aniline polymerization method by modifying our early process [41,42,43]. A solution S1 of 100 mL composed of 0.5 M HCl and 0.4 M ANI was prepared and kept at a controlled temperature of 5 °C. Another solution S2 of 100 mL consisting of 0.5 M HCl, 0.2 M APS, and 0.181 M of metal precursor (either Ni(NO_3_)_2_·6H_2_O or Co(NO_3_)_2_·6H_2_O) was prepared. Under vigorous stirring, S2 was added to S1 at 0.3 L h^−1^ by using a two-syringe infusion pump (KD Scientific, Holliston, MA, USA). The reaction operated for 13 h followed by the solvent removal using a rotavap. The solid polymer product was dried in an oven at 80 °C overnight. The raw materials obtained after this step were referred to as PANI-Ni and PANI-Co. Three other materials were obtained by mixing the initial ones at the ratio PANI-Ni:PANI-Co of 3:1, 1:1, and 1:3 based on the atomic ratio of Ni:Co. These raw materials underwent a thermal treatment under N_2_ in a tubular furnace at 300 °C h^−1^ up to the dwell (50 °C, 1 h) and slowed down to 120 °C h^−1^ toward 900 °C for 6 h. This produces five electrocatalysts based on atomic ratio Ni:Co of 1:0, 3:1, 1:1, 1:3, and 0:1 (it also works in mass because Ni and Co have practically the same atomic weight).

### 2.3. Physicochemical Characterization of the Synthesized Electrocatalysts

X-ray diffraction patterns were obtained on a PANalytical Xpert-PRO diffractometer [40 kV, 20 mA, λ(Cu_Kα1,α2_) = 1.541 Å, Bragg-Brentano mode, 2θ = 10° to 80°]. Thermal behaviors by thermogravimetric analysis (TGA) and differential scanning calorimetry (DSC) were done on SDT Q600 TA Instruments from room temperature to 950 °C (5 °C min^−1^, N_2_ flow of 100 mL min^−1^). Analysis by scanning electron microscopy (SEM) was performed by using a Hitachi S-4800 FEG microscope and energy-dispersive X-ray spectroscopy (EDX) was carried out by using ZEISS EVOHD 15 microscope.

### 2.4. Electrochemical and Electrocatalytic Analysis

A gas diffusion electrode also referred to as carbon paper electrode AvCarb MGL370 (370 μm thickness) was used as support of the prepared electrocatalysts for electrochemical testing at room temperature (25 ± 2 °C). While 0.6 M NaCl (equivalent to 3.5 wt % NaCl) appears as characteristic of seawater, various concentrations are routinely tested to stimulate NaCl-poor (0 to 0.1 M [28]), normal (0.5–0.6 M [27,30]), or NaCl-rich (1 to 2.5 M [31,32,37,39,44]) environment during flow electrolysis where H_2_O is consumed to produce H_2_ and O_2_. Indeed, during continuous seawater electrolysis, chloride ions may accumulate progressively to reach 5.3 M NaCl and accelerate the corrosion and/or favors undesirable chloride oxidation (ClOR) to chlorine/hypochlorite [31]. The present work focuses on one of the most severe conditions of NaCl, that is, 1 M (58 g per L). Therefore, the electrolyte was either 1 M NaOH or 1 M NaOH + 1 M NaCl. The half-cell measurements were performed in a conventional three-electrode setup using a glassy carbon plate of large surface area as the counter electrode and Ag/AgCl/KCl (3 M) as the reference electrode. For calibration of the recorded potentials versus reversible hydrogen electrode (RHE), the conversion was made using E(V vs. RHE) = E(V vs. Ag/AgCl/KCl (3 M)) + 1.02 (calibrating curve obtained in H_2_-saturated electrolyte). The working electrode consisted of a piece of carbon paper electrode cut in 1 cm high by 0.5 cm wide (with sufficient space on top for the electrical wiring with gold) that was cleaned with iPrOH under shaking and dried in an oven. It was followed by the deposition of 10 µL of the prepared catalytic ink by ultrasonic mixing of 260 µL water, 100 µL iPrOH, 40 µL Nafion^®^ suspension, and 8 mg of catalyst. This step results in a loading of 400 µg_catalyst_ cm^−2^. The metal content being ca. 30 wt %, the final loading is 120 µg_metal_ cm^−2^ (the used area was 2 × (1 × 0.5) = 1 cm^2^, not taking into account the three-dimensional structure of the carbon paper electrode). For the commercial Pt/Vulcan at Pt content of 20 wt%, the loading is 80 µg_metal_ cm^−2^. For full-cell experiments in a single compartment cell, the electrode was 2 cm high and 2 cm wide (leaving behind a used area of 2 × (2 × 2) = 8 cm^2^, not taking into account the three-dimensional structure of the carbon paper electrode). Subsequently, a volume of 80 µL was drop-casted onto each face to reach the same loading as half-cell experiments. The potentiostat was the AUTOLAB PGSTAT 204 (Metrohm, Netherlands). All the reported potentials were corrected by the ohmic (iR) drop where the cell resistance of 1.1–1.7 Ω was determined by the potentiostatic electrochemical impedance spectroscopy (EIS) performed at an overpotential of about 100 mV relative to the onset potential (10 mV amplitude, 100 kHz–100 mHz frequency range, 10 points per decade). The durability/stability tests were performed at 100 mA in electrolysis cell mode.

### 2.5. Quantification of Oxidized Chloride Species by Zero Current Potentiometry

After the bulk electrolysis of 1 M NaOH + 1 M NaCl (40 mL), 10 mL HCl (37%), and 620 mg KI were added to chemically generate Cl_2_ (Cl^−^ + ClO^−^ + 2H^+^ → Cl_2_ + H_2_O) that will further produce I_2_ by the quantitative redox reaction 2I^−^ + Cl_2_ → I_2_ + 2Cl^−^. Immediately after, 20 mL of the mixture was taken and titrated by a solution of 1 mM of sodium thiosulphate (2S_2_O_3_^2−^ + I_2_ → 2I^−^ + S_4_O_6_^2−^) using the electroanalytical method of zero current potentiometry to better determine the equivalence point (see Section 3.4 for more details).

## 3. Results and Discussion

### 3.1. Methodology for the Engineering of the Heterogeneous Electrocatalysts

**Figure 1:**
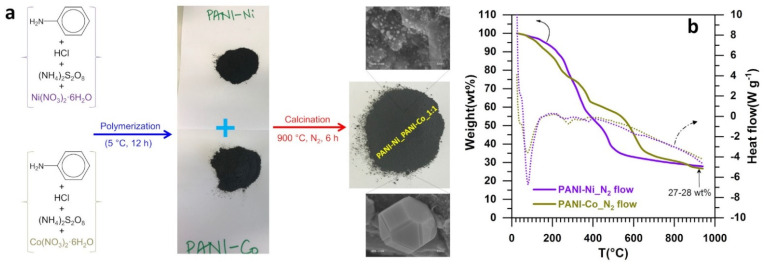
(**a**) Sketch of the engineered approach to produce heterogeneous and self-supported electrocatalysts; (**b**) TGA-DSC profiles the starting materials before any thermal treatment.

The engineering strategy adopted in this work was to enable the simultaneous formation of individual chalcogenides of nickel and cobalt onto a carbon-nitrogen-sulfur nanostructured network by directly using the chemical precursors instead of employing sulfur powder as a reactant. Indeed, we hypothesis that this heterogeneous structure will be beneficial for targeting simultaneously HER and OER by inhibiting any electrochemical processes associated to the presence of NaCl. This simplified design principle could potentially contribute to the cost reduction of new generation seawater electrolyzers where the anode electrocatalyst is not different from that of the cathode. The implemented methodology is sketched in Figure 1a. The separate oxidative aniline (precursor of C and N) polymerization in HCl solution in the presence of either Ni(NO_3_)_2_ or Co(NO_3_)_2_ (precursors of Ni, Co, and N) and triggered by ammonium persulfate (precursor of N and S) was followed by the solvent removal (rotovap) to produce the polymerized materials of PANI-Ni and PANI-Co. Then, in order to study the effect of the composition, both raw materials were mixed to build up five compositions according to the ratio PANI-Ni:PANI-Co of 1:0, 3:1, 1:1, 1:3, and 0:1 based on atomic ratio of Ni:Co after calcination. We have chosen here to not introduce both precursors of nickel and cobalt simultaneously, as our preliminary results [41] have shown that this could result in the formation of single phase of nickel-cobalt. This single phase would cancel the targeted benefit of having the chalcogenides of nickel and cobalt separately within the same electrocatalyst.

Aiming to choose the best temperature for the thermal treatment of both constituents (preliminary data with only Ni in [42]), we conducted TGA-DSC analysis to guide the selection under N_2_. The thermal behavior is displayed in Figure 1b. The evaporation of the adsorbed water molecules or those trapped between the polymer chains occurs below 150 °C [45,46,47,48]. According to the literature, the elimination of the residual doping agent plus the decomposition of smaller fragments of polyaniline happen up to 350 °C, while the main chains of polymer are degraded above 350 °C [47,48] or even 400 °C in the presence of a metallic cation [42]. Here, the extension of these phenomena up to nearly 700 °C is essentially due to the nature of the used gas, that is, N_2_ instead of air where the entire carbon structure of the polyaniline would have been mineralized from ca. 500°. The above ranges of temperature under N_2_ regulate the rearrangement of the different atoms into bonds of carbon-carbon and carbon-heteroatom. After having conducted several control experiments and building on our preliminary study with PANI-Ni (no significant change of the material characteristics and performance towards HER and OER in KOH electrolyte for treatment at 900–1000 °C) [42], 900 °C was chosen for the calcination presented herein. Finally, the metal content of about 30 wt % is in agreement with the theoretical expectation by considering the mineralization yield of our protocol. It is known that during the calcination, the rearrangement of the different atoms from the starting materials produces the bonds such as carbon-carbon (C-C, C=C, C-H) and carbon-nitrogen (pyridinic-N, pyrrolic-N, and graphitic-N) within a porous-like structure that contribute to the performance by the electrical conductivity, the electrocatalytic kinetics and the high number of active sites [42,49,50,51,52,53,54]. Our previous and extensive study (SEM, EDX, TGA-DSC, XPS, BET, XRD, etc.) of polyaniline vs. polyaniline-nickel materials [42] revealed that the thermal treatment produces different types of carbon structures that collectively contribute to the electrocatalysis.

### 3.2. Physicochemical Characterization of the Materials

**Figure 2:**
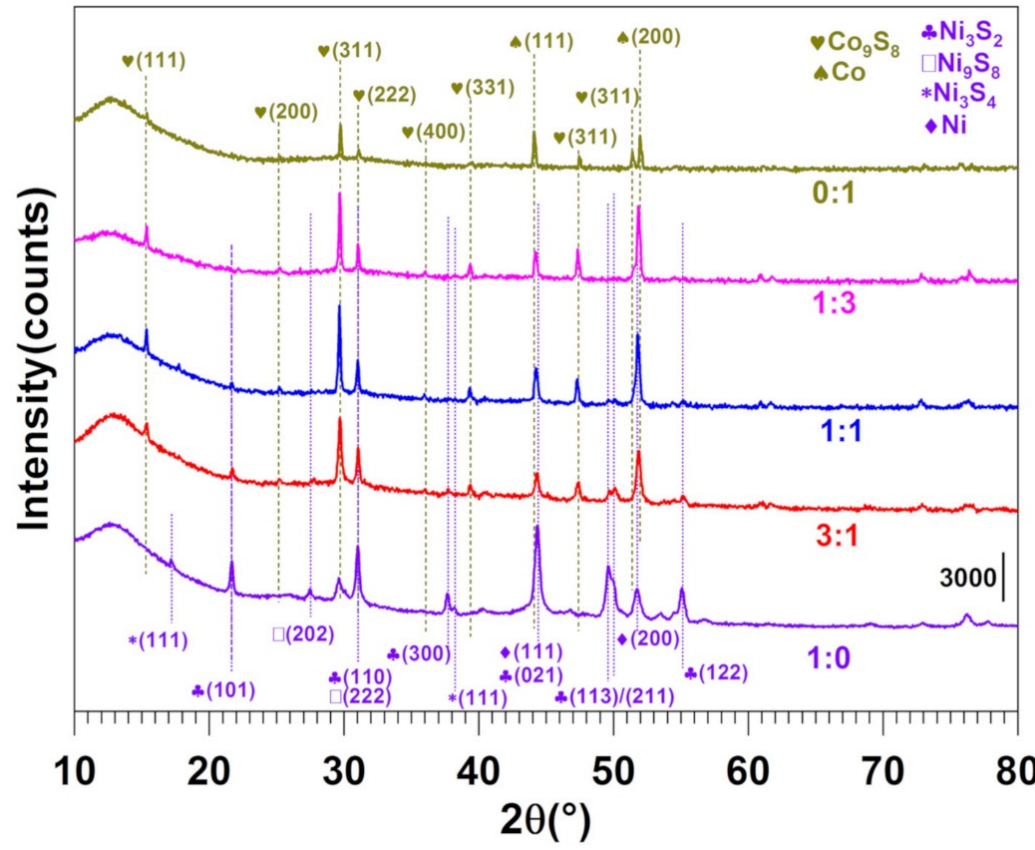
XRD patterns for electrocatalysts obtained for PANI-Ni:PANI-Co ratios of 1:0, 3:1, 1:1, 1:3, and 0:1 based on atomic ratio of Ni:Co after calcination.

To verify our hypothesis on the simultaneous formation of individual chalcogenides of nickel and cobalt onto a carbon-nitrogen-sulfur nanostructured network by the above methodology, we utilized the XRD characterization (Figure 2). For the sample 0:1 (100% Co), the diffraction pattern can be principally indexed by considering a mixture of two phases: metallic face centered cubic (fcc) Co (JCPDS 15–0806) and a cubic phase of Co_9_S_8_ (JCPDS 86-2273), which means that we succeeded in obtaining CoS_x_ for x = 0 and x = 8/9. For the sample 1:0 (100% Ni), the different Bragg peaks demonstrate the mixture of fcc Ni (JCPDS 03-1051) and different nickel sulfide phases, namely Ni_3_S_2_ (JCPDS 44-1418), Ni_3_S_4_ (PDF 01-076-1813) and Ni_9_S_8_ (PDF 01-078-3209). For both monometallic-based materials (broad peak at ca. 12° belongs to the support), no trace of any metal carbide or nitride was detected in the diffraction pattern. Only the Bragg peak located at 2θ = 29.6° for 1:0 could not be attributed to any of sulfide, carbide, nitride, oxide, hydroxide, carbonate, nitrate, or sulfate phases of Ni. Regarding the other samples of intermediate overall composition (3:1, 1:1, and 1:3), they are obviously constituted by a mixture of the phases from the monometallic materials. The Co_9_S_8_ phase remains predominant in the XRD patterns of all the intermediate samples, nickel sulfide phases being mainly detected in the 3:1 sample. No proof of the presence of mixed Ni-Co nanoparticles can be clearly evidenced, which is in agreement to our initial expectations for the synthesis of heterogeneous materials composed of NiS_x_ (x = 0, 2/3, 8/9, and 4/3) and CoS_x_ (x = 0 and 8/9).

**Figure 3:**
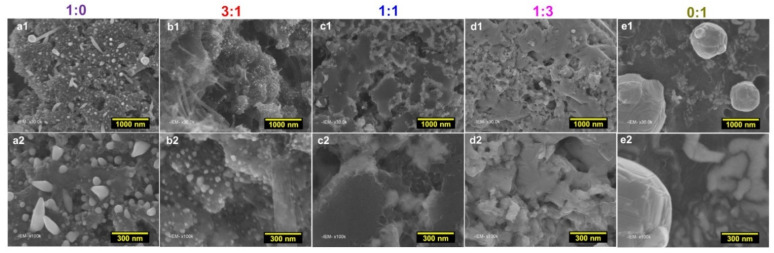
SEM images (at different magnifications) of the as-fabricated electrocatalysts for PANI-Ni:PANI-Co ratios of 1:0 (**a1**,**a2**), 3:1 (**b1**,**b2**), 1:1 (**c1**,**c2**), 1:3 (**d1**,**d2**), and 0:1 (**e1**,**e2**) based on atomic ratio of Ni:Co after calcination.

We next performed qualitative observations by SEM to gain insights on the morphology of the particles, Figure 3. The material obtained for 1:0 (100% Ni) shows a particular morphology, which was previously explained by the vapor-liquid-solid (VLS) mechanism that involves the simultaneous generation of a vapor of nickel-sulfur so that the super-saturation and nucleation at the liquid/solid interface leads to an axial crystal growth [55]. While the sample 0:1 displays bigger size of particles, the intermediate compositions show two behaviors. For a higher content of Ni (3:1), well-defined nanoparticles can be observed while for 1:1 and 1:3, the elements were self-assembled into a nanostructured network. This morphology might expose more active sites to the electrocatalytic reactions and reduce the charge transfer resistance associated to the electron transfer processes. Combined to the previous XRD analysis, this set of microscopy data confirm that the designed methodology allows synthesizing different types of materials that could enable to target different electrocatalytic performance.

**Figure 4:**
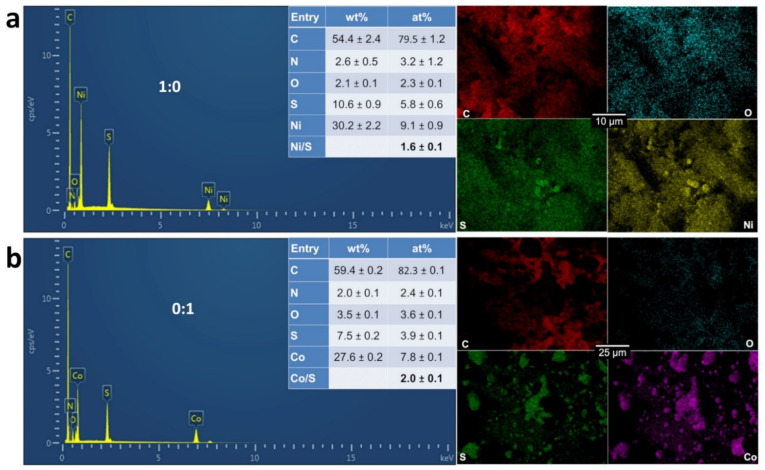
EDX spectra and the corresponding EDX maps of the as-synthesized monometallic electrocatalysts for PANI-Ni:PANI-Co ratios of: 1:0 (**a**), and 0:1 (**b**) based on atomic ratio of Ni:Co after calcination.

To provide quantitative information, we further characterized the materials by coupling SEM to EDX. We were particularly interested by the distribution of the different elements of Ni, Co, C, N, S, and O within the materials. We firstly analyzed single metal materials 1:0 (100% Ni, Figure 4a) and 0:1 (100% Co, Figure 4b) to establish a correlation between EDX mapping and the initial characterization by XRD. The first observation that O signal does not overlap with that of Ni or Co fully rules out any substantial formation of oxide, hydroxide, or oxyhydrooxide phases. Furthermore, the absence of any peak of Co for the 1:0 sample confirms that the XRD peak at 2θ = 29.6° (Figure 2) is not a contamination by the cobalt during the synthesis. The mapping of Figure 4a between Ni and S suggests that those elements are together, which is also true for Figure 4b between Co and S. The overall atomic ratios (Ni/S = 1.6 and Co/S = 2) suggest that the amount of a single metallic phase is more important for 0:1 than 1:0. However, this method cannot differentiate the metals (Ni, Co) from the chalcogenides NiS_x_ (x = 2/3, 8/9 and 4/3) and CoS_x_ (x = 8/9) as revealed by XRD.

**Figure 5:**
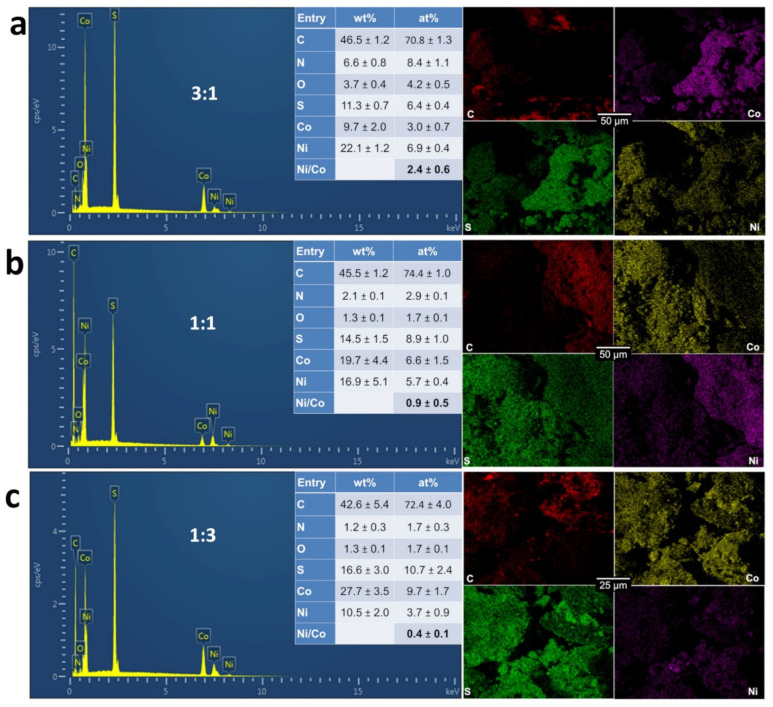
EDX spectra and the corresponding EDX maps of the as-synthesized bimetallic electrocatalysts for PANI-Ni:PANI-Co ratios of: 3:1 (**a**), 1:1 (**b**), and 1:3 (**c**) based on atomic ratio of Ni:Co after calcination.

SEM-EDX results of Figure 5a–c confirm that both Ni and Co are present within the same material as previously observed by EDX. Additionally, we did not observe any significant amount of oxygen nor a correlation between O signal and those of the metals. The mapping images for the samples 3:1 and 1:1 (Figure 5a,b) demonstrate that, in the same block of material, nickel and cobalt are not at the same position. This is exactly what we were targeting in order to maximize the multifunctional character by having different phases in the same electrocatalytic material. This is in agreement with the previous characterization by XRD of distinct phases of NiS_x_ (x = 0, 2/3, 8/9 and 4/3) and CoS_x_ (x = 0 and 8/9). The quantified atomic ratio of Ni/Co is close to the theoretical targets of 3, 1, and 3 for 3:1, 1:1, and 1:3. Furthermore, the EDX mapping for N element in Figures S1–S5 do no highlight any significant overlapping between N and Ni or Co. This would mean that a metal-nitrogen structure was not clearly obtained. However, the calcination likely triggers the formation of carbon-nitrogen bonds (pyridinic-N, pyrrolic-N, and graphitic-N) [42] and the metal-support interaction could cooperatively amplify the electrocatalytic properties. Taken together, these results demonstrate that we succeeded in engineering a strategy to enable the simultaneous formation of individual chalcogenides of nickel (NiS_x_, x = 0, 2/3, 8/9, and 4/3) and cobalt (CoS_x_: x = 0 and x = 8/9) onto a carbon-nitrogen-sulfur nanostructured network by a direct use of chemical precursors instead of employing sulfur powder as a reactant. Dou et al. [56] observed that the nitrogen doping of Co_9_S_8_/graphene leads to high electrocatalytic performance towards OER in alkaline media by tuning the electronic properties of Co_9_S_8_ and the support. Therefore, herein, the presence of neighboring species of NiS_x_ (x = 0, 2/3, 8/9, and 4/3) is expected to produce more cooperative actions to decrease the overpotential during both OER and HER. To investigate such a hypothesis, we aimed in the next section to probe the electrocatalytic performance of this new heterogeneous material.

### 3.3. Electrocatalytic Performance in Half-Cell

**Figure 6:**
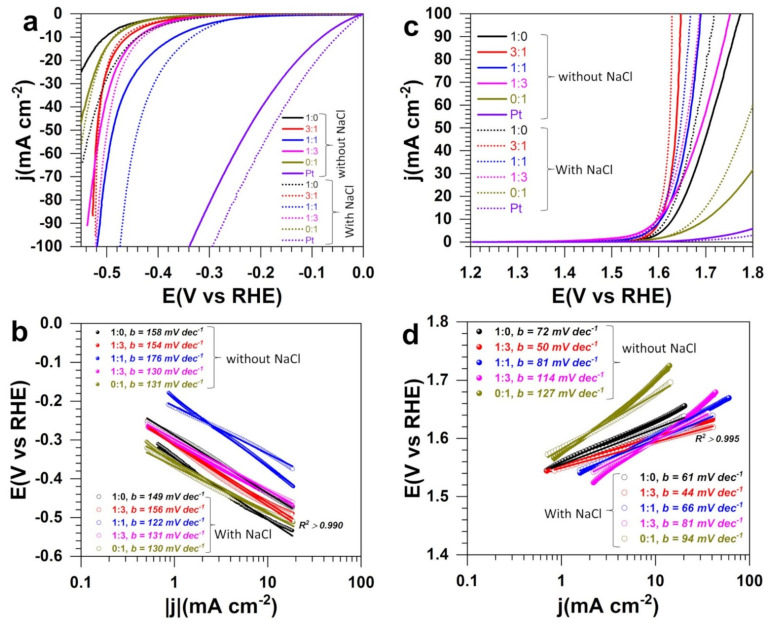
Three-electrode experiments: Polarization curves at room temperature using the as-fabricated electrocatalysts (both at anode and cathode) for PANI-Ni:PANI-Co ratios of 1:0, 3:1, 1:1, 1:3, and 0:1 based on atomic ratio of Ni:Co after calcination. (**a**) HER at 0.005 V s^−1^ in 1 M NaOH in the absence (solid) and presence (dotted) of 1 M NaCl. (**b**) Tafel plots for HER. (**c**) OER at 0.005 V s^−1^ in 1 M NaOH in the absence (solid) and presence (dotted) of 1 M NaCl. (**d**) Tafel plots for OER. Pt refers to commercial electrocatalyst Pt/Vulcan. The support (blank) was a carbon paper electrode of 2 cm high and 2 cm wide—i.e., 8 cm^2^ for both external surfaces.

The electrocatalytic performance towards both HER and OER was first accessed in half-cell configuration (three-electrode setup). Figure 6a–c shows the obtained linear sweep voltammetry (LSV) curves recorded at 5 mV s^−1^ in terms of polarization curves (Figure 6a,c) and Tafel plots (Figure 6b,d). The general observation is that NaCl does not decrease the efficiency of the electrocatalysts, rather, we can observe an amplification. This finding is in agreement with earlier reports by other groups [23,26,27,37,39,44]. Park et al. [29] observed that at Ni-doped FeOOH materials, OER performance decreases significantly by examining 1.0 M KOH and 1.0 M KOH + 0.5 M NaCl electrolytic solutions. Assuming the absence of ClOR interference, this is counter intuitive because the solution 1.0 M KOH + 0.5 M NaCl should have a better ionic conductivity to better drive the electrocatalysis. Yu et al. [44] studied the performance of S-(Ni,Fe)OOH catalyst in 1 M KOH + 1 M NaCl and found that it is not so different from that in 1 M KOH + 0.5 M NaCl, confirming that 1 M NaCl can be a relevant condition to anticipate a potential increase of the NaCl concentration during the electrolysis [31,32].

The extracted quantitative data are reported in Table 1. For HER in 1 M NaOH + 1 M NaCl, the potential required to reach the metric current density of j = −10 mA cm^−2^ is −0.440, −0.466, −0.335, −0.430, and −0.488 V vs. RHE for 1:0, 3:1, 1:1, 1:3, and 0:1, respectively (−0.3 V vs. RHE at commercial Pt/Vulcan). The same trend is observed for the Tafel slopes of 149, 156, 122, 131, and 130 mV dec^−1^ (93 mV dec^−1^ at commercial Pt/Vulcan, Figure S6a), which is significantly lower compared to 1 M NaOH alone. These Tafel slopes that account for the reaction mechanism suggest that HER is limited by the adsorption step of the water molecules (2H_2_O + 2e^−^ → H_2_ + 2OH^−^), known as the Volmer step as mechanistically explained by Equations (6)–(8) [36,57,58]. Hence the heterogeneous structure promotes the adsorption and the electron transfer.

(6) $$\mathrm{Volmer}\;\mathrm{step}:\;H_{2}O+*+e^{-}\;\to \;H*+{\mathrm{OH}}^{-},\;b=\frac{2.3\mathrm{RT}}{\alpha F}=118.2\;\mathrm{mV}\;{\mathrm{dec}}^{-1}\;\mathrm{at}\;25\;{}^{\circ}C,\;$$

(7) $$\mathrm{Heyrovsky}\;\mathrm{step}:\;H_{2}O+H*+\;e^{-}\;\to \;H_{2}+{\mathrm{OH}}^{-}+*,\;b=\frac{2.3\mathrm{RT}}{\left(1+\alpha \right)F}=39.4\;\mathrm{mV}\;{\mathrm{dec}}^{-1}\;\mathrm{at}\;25\;{}^{\circ}C,$$

(8) $$\mathrm{Tafel}\;\mathrm{step}:\;H*+H*\;\to \;H_{2}+2*,\;b=\frac{2.3\mathrm{RT}}{2F}=29.5\;\mathrm{mV}\;{\mathrm{dec}}^{-1}\;\mathrm{at}\;25\;{}^{\circ}C,$$

where b is the Tafel slope (mV dec^−1^), α is the symmetry coefficient (typically, α = 0.5), F is the Faraday constant (96 485 C mol^−1^), R is the ideal gas constant (8.314 J K^−1^ mol^−1^), and T is the absolute temperature (273.15 + °C), H* is hydrogen adsorbed on the active site of electrode surface and * represents the active site of catalysts.

The composition at equal quantity of Ni and Co gives the best performance while the compositions 3:1 and 1:3 tend towards the same overpotential at j = −100 mA cm^−2^. The performance obtained here is lower than the overpotential of 165 mV achieved at 10 mA cm^−2^ for HER in seawater at integrated hierarchical sandwich-like NiCoN|NixP|NiCoN electrocatalysts [59], keeping in mind that these categories of open-pore foam-type electrodes of three-dimensional materials may lead to an overestimation of the HER performance indicators [60]. The enhancement of the electrocatalytic properties for the bimetallic electrodes results from the cooperative action of both chalcogenides of nickel (NiS_x_, x = 0, 2/3, 8/9, and 4/3) and cobalt (CoS_x_: x = 0 and x = 8/9) and the metal-support interaction that facilitate the adsorption of water molecules and the electron transfer as Equation (6) was found to be the limiting step of the HER mechanism. Moreover, the open morphology of the bimetallic materials might expose more active sites to the electrocatalytic reactions and contribute to the observed high activity.

Table S1 (Supplementary Materials) gathers the comparative performance of recently reported relevant electrocatalysts for OER in alkaline simulated seawater electrolyte. From Figure 6c and Table 1 of OER, the achieved performance of 1.6 V vs. RHE with the bimetallic electrocatalysts is competitive to reported systems where the metric current density of j = 10 mA cm^−2^ is obtained in the potential range of 1.5–1.7 V vs. RHE [23,26,27,29,30,37,61,62]. The positive impact of NaCl on the performance is confirmed by a reduced Tafel slope of 44–81 mV dec^−1^, which is significantly lower than the commercial Pt/Vulcan (196 mV dec^−1^, Figure S6b). Our idea of combining both metals of Ni and Co to engineer a heterogeneous material as confirmed by XRD and SEM-EDX analysis is finally a great strategy, because it can be observed in Figure 6 that the bimetallic system outperforms the single metal-based electrodes—particularly when a high current density is demanded. The enhancement of OER performance is related to the improvement of the ionic conductivity of the solution, the co-existence of both chalcogenides of nickel (NiS_x_, x = 0, 2/3, 8/9, and 4/3) and cobalt (CoS_x_: x = 0 and x = 8/9), the metal-support interaction and the open morphology of the bimetallic materials that exposes more active sites to facilitate the adsorption of hydroxyl anions and the electron transfer (4OH^−^ → O_2_ + 2H_2_O + 4e^−^). Based on the thermodynamic prediction of Equations (2) and (3) with E°(ClO^−^/Cl^−^) = 1.72 V_RHE_, and E°(O_2_/OH^−^) = 1.23 V_RHE_ (even though this does not necessarily reflect the reality because all the involved species are not present in the beginning of the experiments [57]), the onset potential below 1.5 V_RHE_ suggests that only OER is occurring. However, it is possible that at a higher current density, ClOR could compete and reduce the OER’s faradaic efficiency. Therefore, an electroanalytical investigation is required to clarify that. It is the aim of the next set of experiments.

### 3.4. Electrocatalytic Performance in Electrolysis Cell and Electroanalytical Quantification

**Figure 7:**
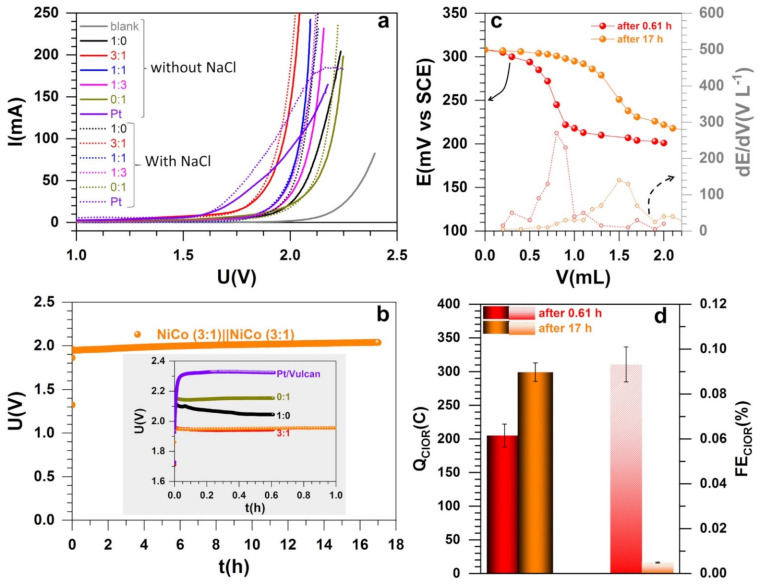
Two-electrode experiments: Water splitting realized at room temperature using the as-fabricated electrocatalysts (both at anode and cathode) through the ratio PANI-Ni:PANI-Co of 1:0, 3:1, 1:1, 1:3, and 0:1 based on atomic ratio of Ni:Co after calcination. (**a**) Polarization curves recorded at 0.005 V s^−1^ in 1 M NaOH in the absence (solid) and presence (dotted) of 1 M NaCl. (**b**) Chronopotentiometry curve recorded at 100 mA using the electrocatalyst corresponding to PANI-Ni:PANI-Co of 3:1. (**c**) Two-electrode potentiometry at zero current for the iodide titration (20 mL: electrolysis solution + HCl + KI) by thiosulphate (1 mM). (**d**) Quantified oxidized chloride species by the potentiometry assays in terms of charge (left *y*-axis) and faradaic efficiency (right *y*-axis). Pt/Vulcan refers to commercial electrocatalyst. The support (blank) was a carbon paper electrode of 2 cm high and 2 cm wide—i.e., 8 cm^2^ for both external surface.

We finally tested the synthesized materials in a single electrolysis cell in the absence and presence of a relevant amount of NaCl (as alkaline seawater [23,24,25,26,27,28,29,30,37]). Figure 7a shows the polarization curves recorded at the quasi-steady state scan rate of 0.005 V s^−1^. Table S2 resumes the comparative performance of recently reported relevant electrocatalysts for the overall water splitting in an alkaline simulated seawater electrolyte where the metal loading is significantly high (1–16 mg cm^−2^ [25,29,32,44,62] vs. 0.4 mg cm^−2^ herein) in order to maintain satisfactory performance. Herein, the electrolysis starts at a cell voltage of about 1.6 V, which is 0.15 V more positive than the thermoneutral point of 1.45 V at 25 °C (never 1.23 V) that takes into account the entropy change during the water splitting [6,63,64]. The data of interest are gathered in Table 2. Except the material 1:1 where the performance at higher current decreases slightly, the general trend is up, and confirms the previous studies [23,61]. Given the half-cell performance of the material 1:1 for HER, a much lower cell voltage was expected for the whole seawater splitting. Such a discrepancy leading sometimes to an inversion of the trends between the half-cell (typically on small size electrode, herein 1 cm^2^) and the whole cell (5–25 cm^2^, herein 8 cm^2^) is recurring in the literature [40,65]. There is a call in the research community to operate under more realistic conditions—e.g., employing gas diffusion electrode (GDE)—such as those employed herein as support (instead of glassy carbon of <1 cm^2^) before claiming any superior electrocatalysts to avoid any large gap in real electrolyzers [40]. Here, while the electrocatalyst synthesis and the catalytic ink preparation did not change between the two sets of experiments, the possible explanation is the change of the electrode–electrolyte interface induced by the scaling of the geometric surface area. This often leads to a drastic modification of the environment of the active sites. The 3:1 composition has the best performance in polarization curves was selected (with the monometallic materials) for durability tests and quantitative analysis of the reaction selectivity.

Figure 7b shows the chronopotentiometry curves recorded at 100 mA where the bimetallic maintains a cell voltage below 2 V after 17 h of reaction. After the bulk electrolysis, we implemented an electroanalytical method of zero current potentiometry to better determine the equivalence point (as detailed in Section 2.5). Equivalent methods are reported with a simple titration or UV–vis spectrometry [24,27,37,39]. Figure 7c shows the typical behavior where the equivalence point corresponds to the maximum of dE/dV. The observed phenomenon (potential-volume) does not result from electrochemical reactions at the electrode–electrolyte interface, but from a redox reaction in solution leading to a modification of the chemical composition of the solution, hence the change of the Nernst potential. This approach is specific to titrations involving fast systems as herein since the potential at zero current is well-defined.

The number of moles of the thiosulphate for the three samples of 1:0, 3:1, and 1:0 after 0.61 h (2200 s) is 0.85 ± 0.07 mmol. For the sample 3:1, after 17 h of reaction, 1.24 ± 0.05 mmol was obtained, which is significantly lower if one hypothesizes a linear increase according to Equation (2). Hence, for the three samples of 1:0, 3:1, and 1:0, after 0.61 h (2200 s), the electrical charge associated to one mole of electron during ClOR is Q_ClOR_ = 205 ± 17 mC after considering the stoichiometry of the involved reactions as well as the electrolysis and titration conditions. For 17 h of electrolysis, the charge associated to ClOR is Q_ClOR_ = 299 ± 13 mC. According to the second law of Faraday, for one mole of electron (Q = I × Δt), the total electrical charge is Q_tot_ = 220 C (0.61 h), and 6121.4 C (17 h). Hence the faradaic efficiency associated to ClOR is FE_ClOR_ = Q_ClOR_/Q_tot_ = 0.09 ± 0.01% after 0.61 h and nearly zero (0.0049 ± 0.0002) after 17 h, as shown in Figure 7d. In other words, there is no significant formation of chemical species from the oxidation of chloride ions. It means that the selectivity towards OER is 100% by considering the two possible pathways expressed by Equations (2) and (3). The present faradaic efficiency outperforms the reported data of 87% [24], 95% [27], and 94% [37] among the most relevant quantified systems.

The possible mechanism for OER at the metal chalcogenides in this alkaline media is thus described by the four elementary steps below (Equations (9)–(12)) [10,36,66]. The first step has a Tafel slope of 118 mV dec^−1^ at 25 °C while the other ones have a Tafel slope of 40 mV dec^−1^ at 25 °C if one considers a symmetry coefficient of α = 0.5. Given the experimental finding of Figure 6d and Figure S6b, the limiting step is the hydroxyl anions adsorption on the active site (*). The reduced Tafel slope for the bimetallic materials 3:1 means that this electrocatalyst provides the optimal synergy for the fast adsorption of OH^−^ on the catalytic surface and the electron transfer.

First electron transfer: OH^−^ + * → OH* + e^−^, (9)

Second electron transfer: OH^−^ + OH* + → H_2_O + O* + e^−^, (10)

Third electron transfer: O* + OH^−^ → OOH* + e^−^, (11)

Fourth electron transfer: OOH* + OH^−^ → O_2_ + H_2_O + e^−^ + *, (12)

## 4. Conclusions

In summary, we report novel electrocatalysts for the comparative study of water electrolysis in a simulated alkaline seawater electrolyte. A two-step method combining the oxidative aniline polymerization and the calcination at 900 °C under N_2_ was implemented to obtain heterogeneous materials. The physical characterization (SEM, EDX, and XRD) shows that this strategy enables the formation of individual chalcogenides of nickel (NiS_x_, x = 0, 2/3, 8/9, and 4/3) and cobalt (CoS_x_, x = 0 and 8/9) onto a carbon-nitrogen-sulfur nanostructured network. Half-cell electrocatalytic measurements for HER and OER show that the presence of NaCl has no impact negative on the kinetics. The potential required to achieve the metric current densities of j = 10, and 100 mA cm^−2^ for OER in the simulated alkaline seawater at the bimetallic electrode is 1.60, and 1.63 V vs. RHE, respectively. This high performance was maintained when the electrode materials were tested in a single compartment electrolysis experiments. The electrolysis starts at a cell voltage of 1.6 V, close to the theoretical expectation of 1.45 V at 25 °C. Stability tests at 100 mA have substantiated the good stability of the bimetallic with only 50 mV voltage change after 17 h. Quantitative analysis revealed that the faradaic efficiency associated to the oxidation of chloride ions is less than 0.1%, that is, nearly 100% efficiency towards OER. The present faradaic efficiency outperforms the data of 87–95% from the literature. The origin of the boosted electrocatalytic performance and selectivity results from the cooperative action of both chalcogenides of nickel (NiS_x_, x = 0, 2/3, 8/9, and 4/3) and cobalt (CoS_x_: x = 0 and x = 8/9), the metal-support interaction and the open morphology of the bimetallic materials that exposes more active sites to facilitate the reactants adsorption and the electron transfer. The results obtained herein contribute towards the engineering of novel and Pt-, Ru-, and Ir-free electrocatalysts for the seawater electrolysis to produce H_2_.

## Figures and Tables

**Table 1:** Required potential to reach |j| = 10 mA cm^−2^ during HER and OER for PANI-Ni:PANI-Co ratios of 1:0, 3:1, 1:1, 1:3, and 0:1 based on atomic ratio of Ni:Co after calcination.

Process	Composition	1:0	3:1	1:1	1:3	0:1
HER	1 M NaOH:E (V vs. RHE)	−0.503	−0.468	−0.369	−0.440	−0.487
	1 M NaOH + 1 M NaCl:E (V vs. RHE)	−0.440	−0.466	−0.335	−0.430	−0.488
	ΔE (mV)	63	2	34	10	1
OER	1 M NaOH:E (V vs. RHE)	1.630	1.604	1.606	1.602	1.701
	1 M NaOH + 1 M NaCl:E (V vs. RHE)	1.617	1.595	1.598	1.597	1.678
	ΔE (mV)	13	9	8	5	23

**Table 2:** Quantitative data from the electrolysis at different electrocatalysts (both at anode and cathode) for PANI-Ni:PANI-Co ratios of 1:0, 3:1, 1:1, 1:3, and 0:1 based on atomic ratio of Ni:Co after calcination. Required voltage to reach 80 mA (equivalent to 10 mA cm^−2^).

Composition	1:0	3:1	1:1	1:3	0:1	Pt/Vulcan
1 M NaOH:U (V)	2.12	1.94	2.03	2.08	2.17	1.98
1 M NaOH + 1 M NaCl:U (V)	2.05	1.93	2.03	2.04	2.15	1.88
ΔU (mV)	70	10	0	40	20	10

## Data Availability

Not applicable.

## Supplementary Materials

The following are available online, Figure S1: EDX maps of the as-synthesized monometallic electrocatalysts for PANI-Ni:PANI-Co ratio of 1:0 based on atomic ratio of Ni:Co after calcination, Figure S2: EDX maps of the as-synthesized monometallic electrocatalysts for PANI-Ni:PANI-Co ratio of 0:1 based on atomic ratio of Ni:Co after calcination, Figure S3: EDX maps of the as-synthesized bimetallic electrocatalysts for PANI-Ni:PANI-Co ratio of 3:1 based on atomic ratio of Ni:Co after calcination, Figure S4: EDX maps of the as-synthesized bimetallic electrocatalysts for PANI-Ni:PANI-Co ratio of 1:1 based on atomic ratio of Ni:Co after calcination, Figure S5: EDX maps of the as-synthesized bimetallic electrocatalysts for PANI-Ni:PANI-Co ratio of 1:3 based on atomic ratio of Ni:Co after calcination, Figure S6: Tafel plots of HER OER from LSV at 0.005 V s^−1^ in 1 M NaOH + 1 M NaCl at the commercial electrocatalyst Pt/Vulcan; Table S1: Comparative performance of recently reported relevant electrocatalysts for OER in alkaline simulated seawater electrolyte; Table S2: Comparative performance of recently reported relevant electrocatalysts for the overall water splitting in an alkaline simulated seawater electrolyte.
