# Supplementary material for: Insights on the Electrocatalytic Seawater Splitting at Heterogeneous Nickel-Cobalt Based Electrocatalysts Engineered from Oxidative Aniline Polymerization and Calcination
Source: Molecules. 2021 Sep 30;26(19):5926. doi: 10.3390/molecules26195926 (PMC8512141; doi:10.3390/molecules26195926)
Supplement: Supplementary file 1 [file molecules-26-05926-s001.zip › molecules-1377393-SI.pdf]

Supplementary Information

Insights on the Electrocatalytic Seawater Splitting at Heterogeneous Nickel-Cobalt based Electrocatalysts Engineered from Oxidative Aniline Polymerization and Calcination

Perla Hajjar[1], Marie-Agnès Lacour[2], Nathalie Masquelez[1], Julien Cambedouzou[1], Sophie Tingry[1], David Cornu[1], and Yaovi Holade[1,*]

1 Institut Européen des Membranes, IEM, UMR 5635, Univ Montpellier, ENSCM, CNRS, Montpellier, France; perla.hajjar@enscm.fr (P.H.), nathalie.masquelez@umontpellier.fr (N.M.), ju-lien.cambedouzou@enscm.fr (J.C.), sophie.tingry@umontpellier.fr (S.T.), david.cornu@enscm.fr (D.C.)

2 ChemLab, ENSCM, Montpellier, France, Cedex 5, France; marie-agnes.lacour@enscm.fr (M.-A.L.)

* Correspondence: yaovi.holade@umontpellier.fr ; Tel.: +33-467-14-92-94

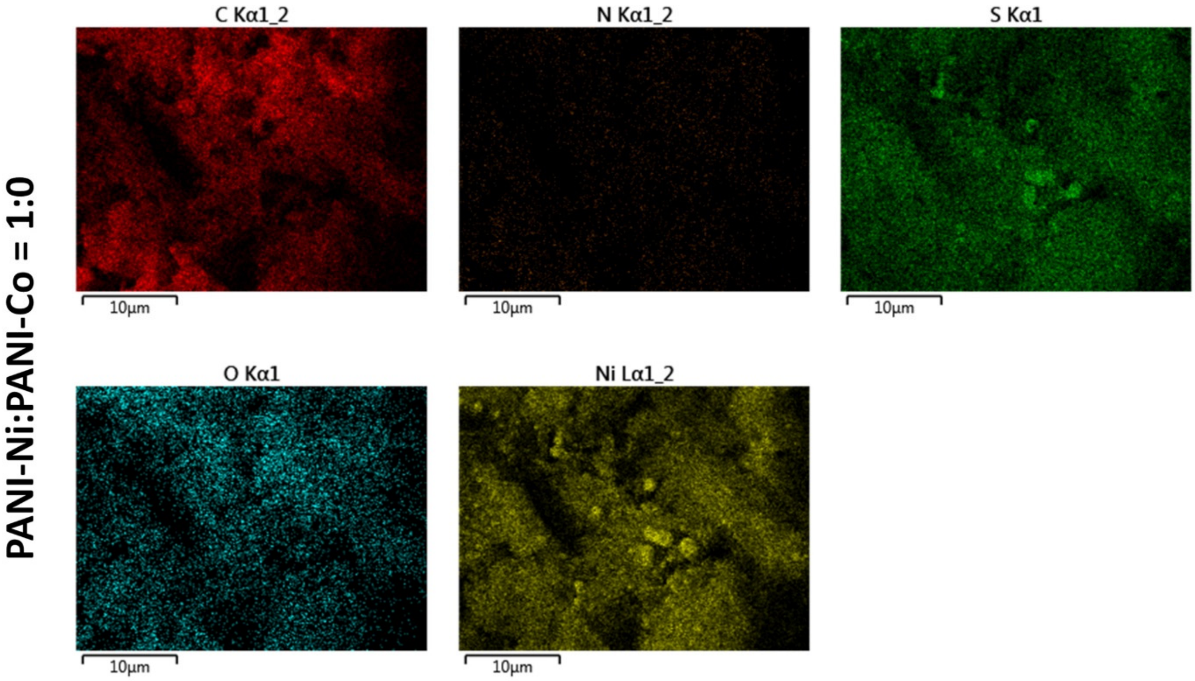

Figure S1. EDX maps of the as-synthesized monometallic electrocatalysts for PANI-Ni:PANI-Co ratio of 1:0 based on atomic ratio of Ni:Co after calcination.

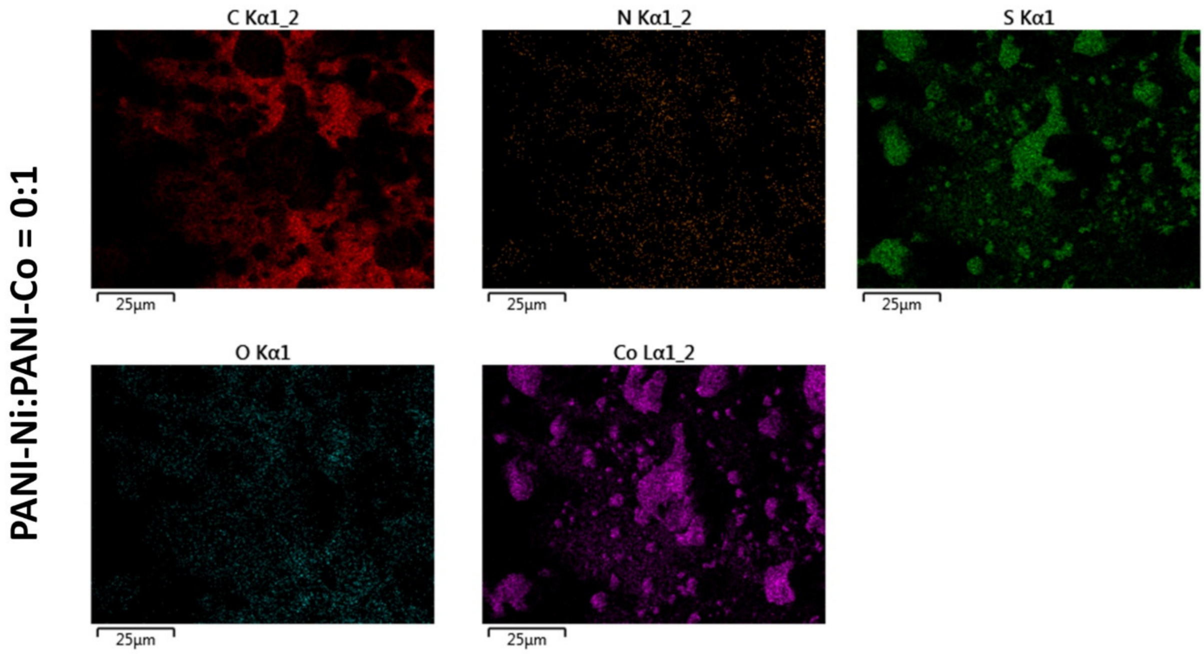

Figure S2. EDX maps of the as-synthesized monometallic electrocatalysts for PANI-Ni:PANI-Co ratio of 0:1 based on atomic ratio of Ni:Co after calcination.

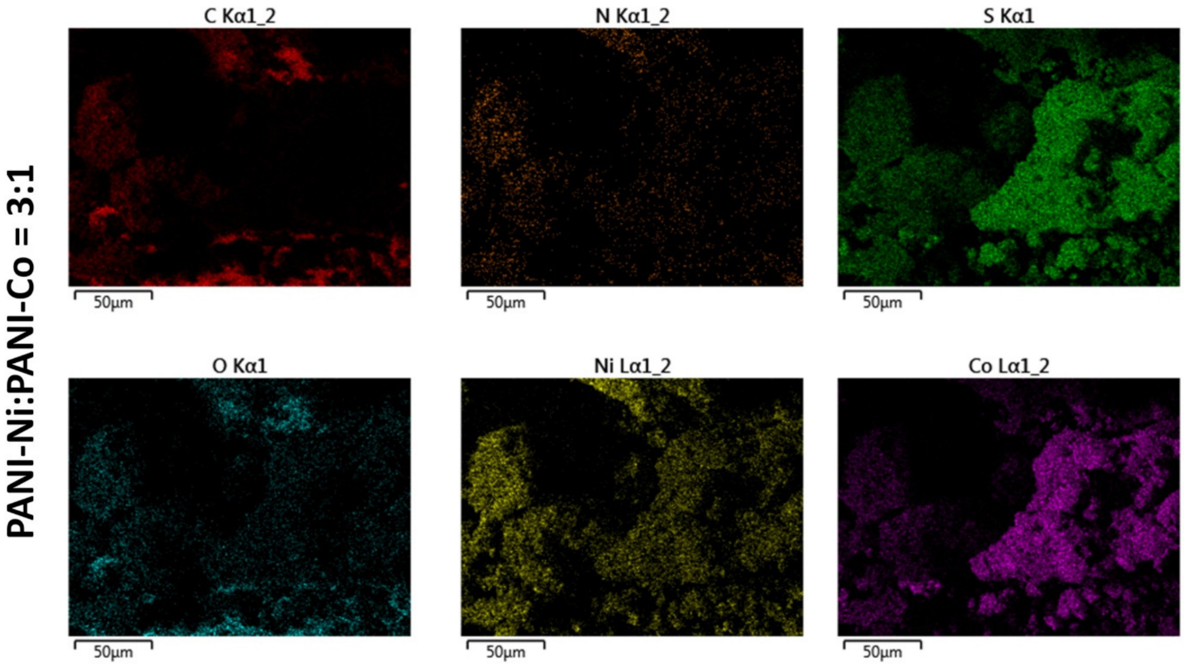

Figure S3. EDX maps of the as-synthesized bimetallic electrocatalysts for PANI-Ni:PANI-Co ratio of 3:1 based on atomic ratio of Ni:Co after calcination.

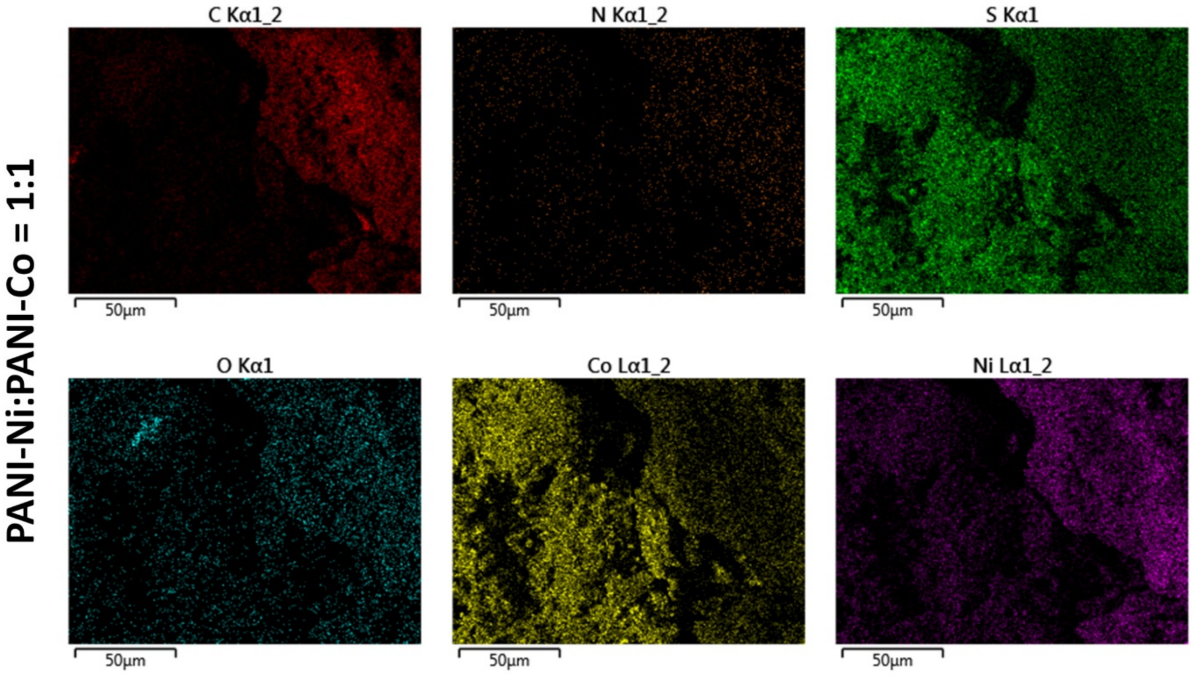

Figure S4. EDX maps of the as-synthesized bimetallic electrocatalysts for PANI-Ni:PANI-Co ratio of 1:1 based on atomic ratio of Ni:Co after calcination.

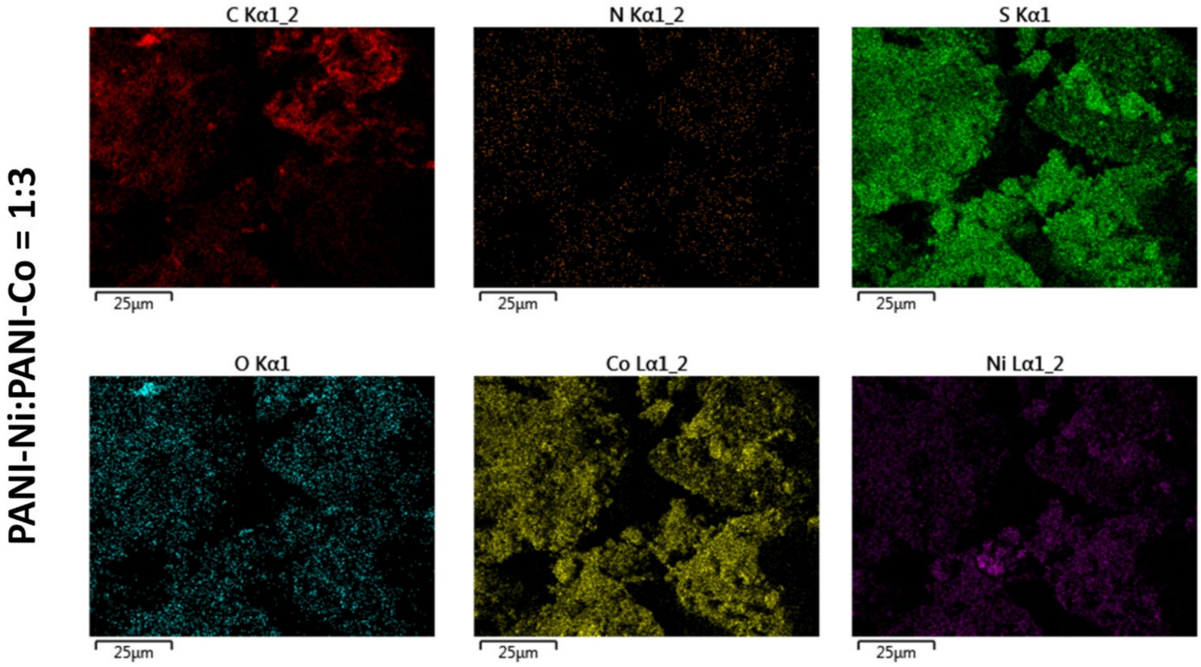

Figure S5. EDX maps of the as-synthesized bimetallic electrocatalysts for PANI-Ni:PANI-Co ratio of 1:3 based on atomic ratio of Ni:Co after calcination.

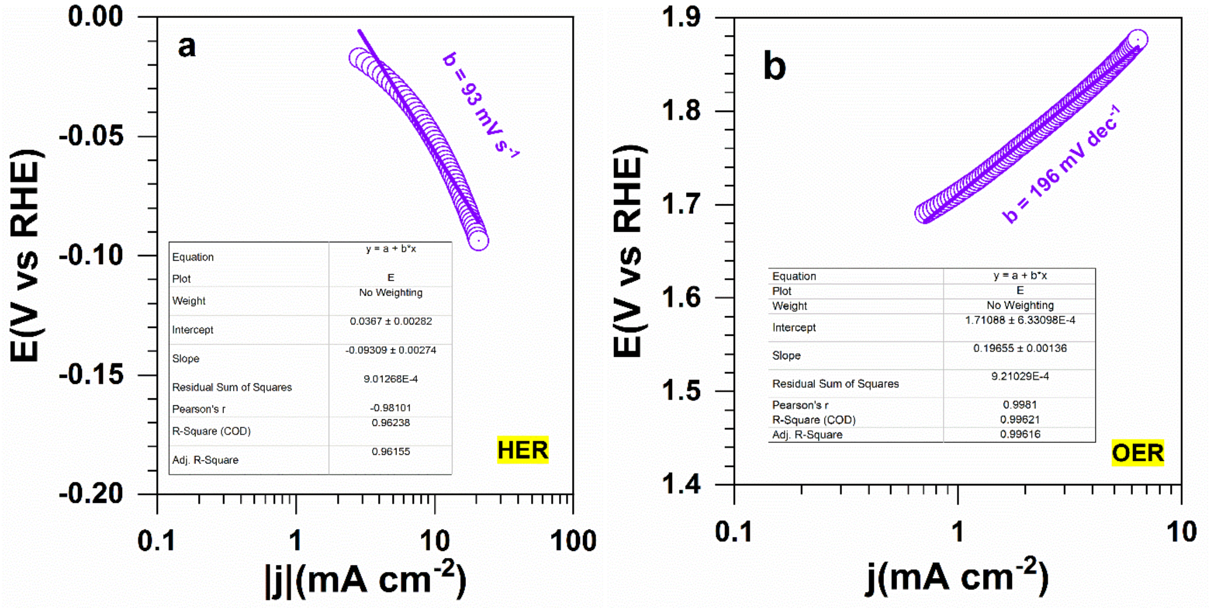

Figure S6. Tafel plots of HER OER from LSV at 0.005 V s^{-1} in 1 M NaOH + 1 M NaCl at the commercial electrocatalyst Pt/Vulcan.

Table S1. Comparative performance of recently reported relevant electrocatalysts for OER in alkaline simulated seawater electrolyte.

Reference	Catalyst	Working electrode Support (area)	Loading (mg cm^{-2})	Electrolyte	Overpotential at 10 or 100 mA cm^{-2} η = E-1.23	Tafel slope (mV dec^{-1})
Herein	NiS_x+CoS_x, x = 0, 2/3, 8/9 and 4/3	Carbon paper (1 cm^2)	0.4	1 M NaOH + 1 M NaCl	η_{10} = 365 mV<br>η_{100} = 365 mV	44
	NiS_x, x = 0, 2/3, 8/9, 4/3				η_{10} = 387 mV<br>η_{100} = 398 mV	61
	CoS_x, x = 0, and 8/9				η_{10} = 448 mV<br>η_{100} > 597 mV	94
	Pt/Vulcan				η_{10} > 670 mV	197
ACS Appl. Energy Mater. **2020,** *3* (8), 7619-7628.[1]	Co-Fe-O-B	GCE	0.1	1 M KOH + 0.5 M NaCl	η_{10} = 294 mV<br>η_{100} = 434 mV	-
ACS Catal. **2021,** *11* (12), 6800-6809 and *Chemsuschem* **2016,** *9* (9), 962-972.[2-3]	NiFe LDH	GCE (0.2 cm^2)	0.1	1 M KOH + 0.5 M NaCl	η_{100} = 359 mV	50
Nat. Commun. **2019,** *10* (1), 5106.[4]	NiMoN@NiFeN	Ni foam	0.3-0.45	1 M KOH + 0.5 M NaCl	η_{100} =286 mV	-
J. Mater. Chem. A **2021,** *9* (15), 9586-9592.[5]	Ni-doped FeOOH	Ni foam	3.41	1 M KOH + 0.5 M NaCl	η_{100} =290 mV	-
Int. J. Hydrogen Energy **2021,** *46* (24), 12936-12943.[6]	NiFe LDH	Carbon cloth	-	1 M KOH + 0.5 M NaCl	η_{10} = 270 mV	52
Energy Environ. Sci. **2020,** *13* (10), 3439-3446. [7]	S-(Ni,Fe)OOH	Ni foam	-	1 M KOH + 1 M NaCl	η_{100} =275 mV	-

For complete literature review (plus HER data) in alkaline simulated seawater electrolyte, reader can refer to a recent Review-article: Liu, G.; Xu, Y.; Yang, T.; Jiang, L., Recent advances in electrocatalysts for seawater splitting. *Nano Mater. Sci.* **2020**, DOI: https://doi.org/10.1016/j.nanoms.2020.12.003.

Table S2. Comparative performance of recently reported relevant electrocatalysts for the overall water splitting in an alkaline simulated seawater electrolyte.

Reference	Catalyst	Electrolyte	Cell voltage at 10 or 100 mA cm^{-2}	Faradaic Efficiency (%)
Herein (multi-functional)	NiS_x+CoS_x, x = 0, 2/3, 8/9 and 4/3	1 M NaOH + 1 M NaCl (room temperature)	U_{10} = 1.93 V	0.09 ± 0.01 in Cl^- oxidation
	NiS_x, x = 0, 2/3, 8/9, 4/3		U_{10} = 2.05 V	-
	CoS_x, x = 0, and 8/9		U_{10} = 2.15 V	-
	Pt/Vulcan		U_{10} = 1.88 V	-
Proc. Natl. Acad. Sci. **2019,** *116* (14), 6624-6629.[8]	Anode = NiFe/NiSx-Ni3 (16 mg cm^{-2} onto Ni foam)<br>Cathode = Ni-NiO-Cr_2O_3 cathode (8 mg cm^{-2} onto Ni foam)	6 M KOH + 1.5 M NaCl (80 °C)	U_{100} = 1.52 V	100% based O_2 quantification
Energy Environ. Sci. **2020,** *13* (6), 1725-1729.[9]	Anode = NiFe LDH (2 mg cm^{-2} onto gold sputtered titanium felt)<br>Cathode = Pt/Vulcan (0.5 mg cm^{-2} onto carbon based GDL (Sigratec BC 25)	0.5 M KOH + 0.5 M NaCl	U_{100} = 1.67 V	100% based O_2 quantification
Nat. Commun. **2019,** *10* (1), 5106.[4]	Anode = NiMoN@NiFeN (0.3-0.45 mg cm^{-2} onto Ni foam)<br>Cathode = NiMoN (0.3-0.45 mg cm^{-2} onto Ni foam)	1 M KOH + 0.5 M NaCl (60 °C)	U_{100} = 1.58 V	2% based H_2:O_2 ratio
J. Mater. Chem. A **2021,** *9* (15), 9586-9592.[5]	Anode = Ni-doped FeOOH (3.41 mg cm^{-2} onto Ti felt)<br>Cathode = Pt/Vulcan (1 mg cm^{-2} onto Carbon cloth)	1 M KOH + 0.5 M NaCl (50 °C)	U_{10} = 1.42 V<br>U_{100} = 1.49 V	-
Int. J. Hydrogen Energy **2021,** *46* (24), 12936-12943.[6]	Anode = NiFe LDH	1 M KOH + seawter	U_{100} = 2.8 V	5% in Cl^- oxidation
Energy Environ. Sci. **2020,** *13* (10), 3439-3446. [7]	Anode = S-(Ni,Fe)OOH (onto Ni foam)<br>Cathode = NiMoN (onto Ni foam)	1 M KOH + 1 M NaCl	U_{10} = 1.45 V<br>U_{100} = 1.63 V	100% based H_2:O_2 = 2:1

References

1. Gupta, S.; Forster, M.; Yadav, A.; Cowan, A. J.; Patel, N.; Patel, M., Highly Efficient and Selective Metal Oxy-Boride Electrocatalysts for Oxygen Evolution from Alkali and Saline Solutions. *ACS Appl. Energy Mater.* **2020,** *3* (8), 7619-7628.
2. Dresp, S.; Dionigi, F.; Klingenhof, M.; Merzdorf, T.; Schmies, H.; Drnec, J.; Poulain, A.; Strasser, P., Molecular Understanding of the Impact of Saline Contaminants and Alkaline pH on NiFe Layered Double Hydroxide Oxygen Evolution Catalysts. *ACS Catal.* **2021,** *11* (12), 6800-6809.
3. Dionigi, F.; Reier, T.; Pawolek, Z.; Gliech, M.; Strasser, P., Design Criteria, Operating Conditions, and Nickel-Iron Hydroxide Catalyst Materials for Selective Seawater Electrolysis. *Chemsuschem* **2016,** *9* (9), 962-972.
4. Yu, L.; Zhu, Q.; Song, S.; McElhenny, B.; Wang, D.; Wu, C.; Qin, Z.; Bao, J.; Yu, Y.; Chen, S.; Ren, Z., Non-noble metal-nitride based electrocatalysts for high-performance alkaline seawater electrolysis. *Nat. Commun.* **2019,** *10* (1), 5106.
5. Park, Y. S.; Lee, J.; Jang, M. J.; Yang, J.; Jeong, J.; Park, J.; Kim, Y.; Seo, M. H.; Chen, Z.; Choi, S. M., High-performance anion exchange membrane alkaline seawater electrolysis. *J. Mater. Chem. A* **2021,** *9* (15), 9586-9592.
6. Lu, J.; Li, C.; Wang, H.; Ji, S.; Wang, X.; Wang, R., How to get to best oxygen evolution behavior from the electrolysis practice of the seawater. *Int. J. Hydrogen Energy* **2021,** *46* (24), 12936-12943.
7. Yu, L.; Wu, L.; McElhenny, B.; Song, S.; Luo, D.; Zhang, F.; Yu, Y.; Chen, S.; Ren, Z., Ultrafast room-temperature synthesis of porous S-doped Ni/Fe (oxy)hydroxide electrodes for oxygen evolution catalysis in seawater splitting. *Energy Environ. Sci.* **2020,** *13* (10), 3439-3446.
8. Kuang, Y.; Kenney, M. J.; Meng, Y.; Hung, W.-H.; Liu, Y.; Huang, J. E.; Prasanna, R.; Li, P.; Li, Y.; Wang, L.; Lin, M.-C.; McGehee, M. D.; Sun, X.; Dai, H., Solar-driven, highly sustained splitting of seawater into hydrogen and oxygen fuels. *Proc. Natl. Acad. Sci.* **2019,** *116* (14), 6624-6629.
9. Dresp, S.; Thanh, T. N.; Klingenhof, M.; Bruckner, S.; Hauke, P.; Strasser, P., Efficient direct seawater electrolysers using selective alkaline NiFe-LDH as OER catalyst in asymmetric electrolyte feeds. *Energy Environ. Sci.* **2020,** *13* (6), 1725-1729.
